# Introduction of rubella-containing-vaccine to Madagascar: implications for roll-out and local elimination

**DOI:** 10.1098/rsif.2015.1101

**Published:** 2016-04

**Authors:** Amy Wesolowski, Keitly Mensah, Cara E. Brook, Miora Andrianjafimasy, Amy Winter, Caroline O. Buckee, Richter Razafindratsimandresy, Andrew J. Tatem, Jean-Michel Heraud, C. Jessica E. Metcalf

**Affiliations:** 1 Department of Epidemiology, Harvard T. H. Chan School of Public Health, Boston, MA, USA; 2 Center for Communicable Disease Dynamics, Harvard T. H. Chan School of Public Health, Boston, MA, USA; 3 Virology Unit and Measles and Rubella WHO National Reference Laboratory, Institut Pasteur de Madagascar, Antananarivo, Madagascar; 4 Department of Geography and Environment, University of Southampton, Southampton, UK; 5 Fogarty International Center, National Institutes of Health, Bethesda, MD, USA; 6 Flowminder Foundation, Stockholm, Sweden; 7 Department of Ecology and Evolutionary Biology, Princeton University, Princeton, NJ, USA; 8 Office of Population Research, Woodrow Wilson School, Princeton University, Princeton, NJ, USA

**Keywords:** rubella, vaccination, Madagascar, congenital rubella syndrome, evaluation of vaccination programmes, Africa

## Abstract

Few countries in Africa currently include rubella-containing vaccination (RCV) in their immunization schedule. The Global Alliance for Vaccines Initiative (GAVI) recently opened a funding window that has motivated more widespread roll-out of RCV. As countries plan RCV introductions, an understanding of the existing burden, spatial patterns of vaccine coverage, and the impact of patterns of local extinction and reintroduction for rubella will be critical to developing effective programmes. As one of the first countries proposing RCV introduction in part with GAVI funding, Madagascar provides a powerful and timely case study. We analyse serological data from measles surveillance systems to characterize the epidemiology of rubella in Madagascar. Combining these results with data on measles vaccination delivery, we develop an age-structured model to simulate rubella vaccination scenarios and evaluate the dynamics of rubella and the burden of congenital rubella syndrome (CRS) across Madagascar. We additionally evaluate the drivers of spatial heterogeneity in age of infection to identify focal locations where vaccine surveillance should be strengthened and where challenges to successful vaccination introduction are expected. Our analyses indicate that characteristics of rubella in Madagascar are in line with global observations, with an average age of infection near 7 years, and an impact of frequent local extinction with reintroductions causing localized epidemics. Modelling results indicate that introduction of RCV into the routine programme alone may initially decrease rubella incidence but then result in cumulative increases in the burden of CRS in some regions (and transient increases in this burden in many regions). Deployment of RCV with regular supplementary campaigns will mitigate these outcomes. Results suggest that introduction of RCV offers a potential for elimination of rubella in Madagascar, but also emphasize both that targeted vaccination is likely to be a lynchpin of this success, and the public health vigilance that this introduction will require.

## 1. Introduction

Rubella is a directly transmitted immunizing infection that usually occurs during childhood and is associated with low morbidity and mortality. Infection of women during early pregnancy can lead to spontaneous abortion, fetal death or children born with congenital rubella syndrome (CRS), which is associated with multiple disabilities that can require lifelong care [1], including hearing impairment, cataracts and congenital heart disease [2].

**Figure 1.**
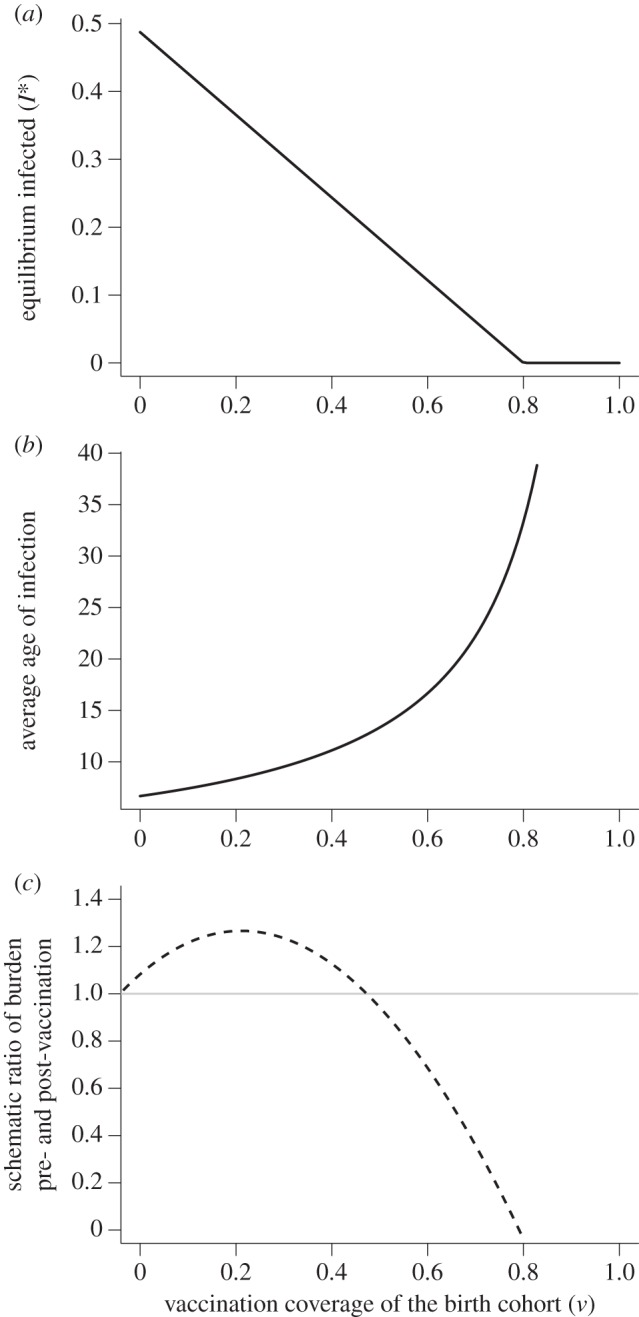
Theoretical expectations for RCV coverage and the burden of CRS. In a classic SIR framework, the dynamics of susceptibles are captured by d*S*/d*t* = *μ*(1 − *v*) − *βSI* − *μS*; and infected by d*I/*d*t* = *βSI* − *gI* − *μI*; where *μ* is the birth and death rate, total population size as taken as *N* = 1, *v* is vaccination coverage of the birth cohort, *g* is the generation time of the infection and the transmission rate is *β* = *R*_0_(*g* + *μ*). To capture rubella dynamics, we set *g* to 18 days^−1^, *R*_0_ = 5, and chose *μ* = 30 per 1000 per year. (*a*) The equilibrium proportion of infected individuals I* (*y*-axis) is defined by I* = *μ*[(1 − *v*)*R*_0_ − 1]/*β* and thus declines with increasing vaccination coverage (*x*-axis). (*b*) Conversely, the average age of infection *A* (*y*-axis) increases, following *R*_0_ = *G*/*A*, where *G* is the inverse of the unvaccinated birth rate, *G* = 1/[*μ*(1 − *v*)]. (*c*) This conjunction of declining incidence but increasing average age of infection has the potential to yield a situation where more cases are occurring in women of childbearing age, even though the total number of cases is declining. As a result, the ratio of the equilibrium burden of CRS in the presence of vaccination relative to the equilibrium burden of CRS if no vaccination has occurred (*y*-axis) may first increase with vaccination coverage relative to the scenario of no vaccination (indicated by the horizontal grey line); eventually declining when incidence is sufficiently low to offset the increase in the average age of infection. (Results in the last panel are hypothetical; exact values will depend on the variance and skew of the distribution of age of infection; as well as pattern of fertility over age). This pattern of increasing CRS burden with increasing vaccination coverage has been called ‘the paradoxical increase of rubella’.

A relatively inexpensive, high efficacy vaccine that provides lifelong immunity to rubella and can easily be combined as measles–rubella (MR), or measles–mumps–rubella (MMR) has been available for 50 years. Although routine rubella vaccination can prevent CRS, inadequate vaccination coverage may actually increase CRS cases by increasing the average age of infection [3]; this occurs because vaccination short of the threshold required for elimination effectively reduces incidence in the population, thus reducing the risk of infection and delaying time to the first infection (figure 1). Consequently, introduction of rubella-containing vaccine (RCV) has been limited globally. Recent efforts for the control and elimination of measles have spurred renewed interest in the potential for rubella control, because the two vaccines are easily combined, and overall measles vaccination coverage levels have been climbing [4]. In addition, the Global Alliance for Vaccines Initiative (GAVI) has recently opened a funding window for rubella vaccination [5]. Madagascar is one of the countries that has successfully applied for this funding.

For all low- and middle-income countries that take advantage of the GAVI funding window, introduction of RCV will likely be shaped by the context of measles childhood immunization efforts. In Madagascar, measles vaccination was introduced into the routine programme in 2004. Coverage increased steeply until political upheavals in 2009, at which point levels fell again; currently, measles vaccination coverage (defined as the number of doses delivered divided by the target population size) today hovers around 85% in Madagascar [6]. One of the perceived possible advantages of introduction of RCV is strengthened investment in measles programmes [7], an important potential public health benefit in Madagascar. However, these benefits must be balanced with broader consequences, including potential negative outcomes of introduction of RCV for the burden of CRS [8]. Birth rates in Madagascar remain relatively high (more than 30 per 1000 per year [9]), and theory suggests that high birth rates are associated with a low burden of CRS in the absence of vaccination [10], making investment for RCV less of a public health priority. Further, with high birth rates, and particularly in the context of high transmission, a very high threshold of vaccination coverage is required to move beyond coverage levels that result in increases in the CRS burden [10]. A final complication is that even where coverage is sufficiently high to ensure a country-scale reduction in the CRS burden, heterogeneous vaccination coverage might result in increases in inequity in the burden of CRS [11,12]. Local rubella extinction allows susceptible women to age into their childbearing years while still retaining susceptibility, thus creating regions at high risk for CRS. Vaccination may alter the flow of infected individuals into these high-risk regions to further elevate the burden of CRS [11].

Understanding key features of the context, and delineating the possible consequences of introduction of RCV into Madagascar has implications for other low-income countries. Many countries across the AFRO region are considering introduction of RCV, and although the WHO position piece on rubella vaccination emphasizes the importance of immunizing women of childbearing age [13], GAVI is not supplying funding beyond that required for introduction into routine childhood immunization programmes. As a result, few countries are investigating this possibility. Understanding the risks associated with a focus on routine vaccination alone, when compared with routine vaccination with investments into regular campaigns, is of considerable public health relevance [14]. It is possible that the particular demographic and vaccination profiles of a given region may be such that routine vaccination will result in serious consequences [15]; and it is important that we consider all possible outcomes when designing public health policy.

Detailed characterization of a country's situation before and after introduction of RCV remains the exception in the current landscape of global health. Using existing data from Madagascar, we are in a position to document the current status of the burden of CRS (thus providing a baseline for further evaluation), identify key surveillance needs and develop a better understanding of the risks in a broader public health context.

Here, we draw on data from a range of sources to ask (i) What is the current epidemiology of rubella in Madagascar? (ii) What is the current context of measles vaccination delivery in Madagascar, and what does this mean for how RCV will be deployed? and (iii) What are the likely consequences of introduction of RCV on both the incidence of rubella and the burden of CRS? We conclude by discussing what these patterns suggest for rubella roll-out more broadly, across low-income countries likely to capitalize on the GAVI initiative.

## 2. Material and methods

### 2.1. Epidemiology of rubella in Madagascar

**Figure 2.**
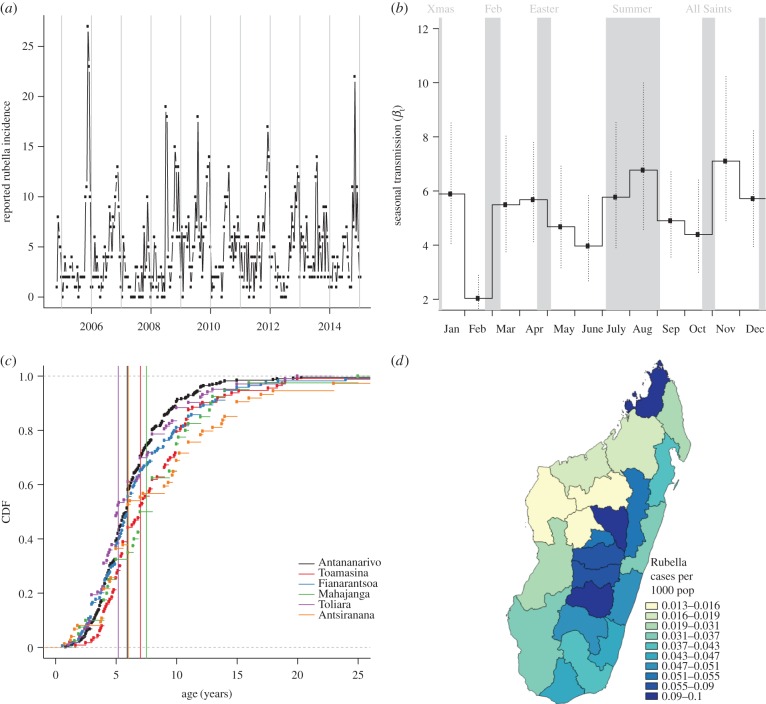
The epidemiology of rubella in Madagascar. (*a*) Reported number of cases through time for the entire country; (*b*) associated estimates of transmission from the TSIR model; (*c*) age distribution of infection, and average age of infection (vertical lines) associated with each of the six provinces and (*d*) Map of incidence in regions of Madagascar across the country with colours showing incidence per 1000 inhabitants. (Online version in colour.)

Rubella data were obtained via the system of general surveillance for measles in Madagascar (figure 2). For all patients presenting with fever, generalized rash, and either cough, coryza or conjunctivitis, a serum specimen was collected, and subsequently tested using standard serological techniques at the WHO national reference laboratory located at the Institut Pasteur of Madagascar in Antananarivo. Samples that were measles IgM negative were tested for rubella IgM antibody. These assays supplied information about the age range and incidence of rubella through time across the six major provinces of Madagascar, and a Time-series Susceptible–Infected–Recovered (TSIR) model was fit to the data (see electronic supplementary material, figure S1). This framework allows for estimation of the fraction of rubella cases that are reported (or reporting rate, here denoted *ρ*), as well as the starting proportion susceptible (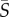), and seasonal fluctuations in rubella transmission (*β*_s_). It would be of considerable interest to compare these patterns to the spatial and temporal trends in incidence of CRS. However, the variety of symptoms associated with CRS and the multiplicity of possible alternative causes of many of these symptoms (such as deafness or cataracts) makes CRS surveillance intractable in low resource settings where serological testing is a rarity; data on CRS are not currently available for Madagascar.

### 2.2. Characterizing measles vaccination in Madagascar

**Figure 3.**
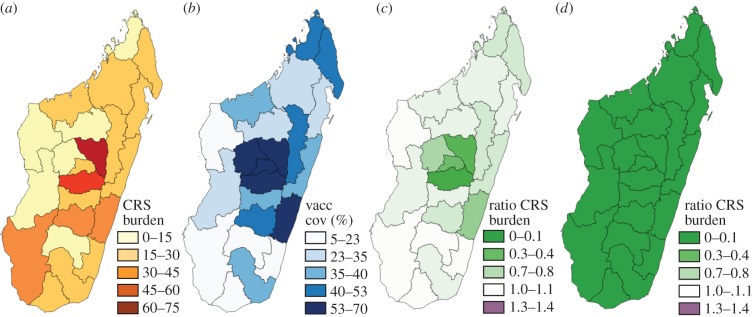
The burden of CRS. (*a*) Simulated deterministic burden of CRS over 30 years in the absence of vaccination for each region; and (*b*) Current routine vaccination coverage values per region (taking the lower conservative scale of routine vaccination coverage). (*c*) Simulated deterministic burden of CRS over 30 years following introduction of routine vaccination only (set at the conservative lower bound) divided by the simulated deterministic burden of CRS obtained in the absence of vaccination (shown in figure 3*a*)). Where the ratio exceeds 1, the introduction of vaccination has resulted in an increase in the burden of CRS. (*d*) Similarly, ratio of the simulated deterministic burden of CRS over 30 years following introduction of routine vaccination with a starting campaign reaching up to age 10, and follow-up campaigns to age 5 occurring every 4 years; all campaigns have a coverage of 60%. The full colour scale for ratios encompasses values more than 1.3 not observed here, but to enable comparison of outcomes with *R*_0_ = 8 (electronic supplementary material, figure S6), and detection of areas where the ratio exceeds 1. See the electronic supplementary material, table S1 for the full set of results. (Online version in colour.)

We consulted the Ministry of Health (MOH) of Madagascar and UNICEF for details on recent measles vaccination coverage. Routine vaccination is provided at most public and private clinics and aims to vaccinate all children around nine months of age. Two additional opportunities for vaccination also exist: (i) Supplementary Immunization Campaigns (occurring in 2007, 2010 and 2013) intended to complement routine vaccination and (ii) mother and child health days, targeting children between 9 and 11 months, which are bi-annual campaigns intended to provide care to the most remote and vulnerable mother and children populations in the country. Vaccination services are provided by the national health authorities (MOH) and non-governmental organizations, primarily UNICEF. We obtained estimates of routine immunization coverage from the MOH for each of the 22 regions (figure 3*b*) of Madagascar in 2014. These data measure the number of doses distributed in each region divided by the target population size, and values range from 52% (in the region of Melaky region) to 92% (in Vakinankaratra region), with an average of 77% (table 1). Deviations in population size as well as the uneven success of dose delivery make these uncertain measures of vaccine delivery [16]. To provide a conservative yet comparable analysis across regions, we therefore used existing administrative data from 2015, which include doses delivered up to 7 July 2015. These estimates range from 5% (in Atsimo-Atsinana) to 70%, (in Vakinankaratra) with an average of 37% (table 1). The reality of measles vaccination coverage in Madagascar is almost certainly greater than these estimates suggest, but these values provide a conservative baseline from which to evaluate areas at risk for increases in the CRS burden in the event of rubella vaccine introduction.

**Table 1.** Measles vaccination coverage in Madagascar. Vaccination coverage in each of the 22 regions, from the 2014 administrative coverage estimates (i.e. doses delivered divided by target population size); and also showing the early 2015 coverage, taken as a conservative lower boundary, as this reflects reports on doses delivered only up until June of 2015, and is accordingly likely an underestimate of doses delivered.

regions	2014 admin coverage	early 2015 admin coverage
Analamanga	0.77	0.65
Itasy	0.67	0.65
Haute matsiatra	0.81	0.48
Amoron'i mania	0.77	0.39
Sofia	0.91	0.25
Alaotra-Mangoro	0.73	0.45
Vatovavy Fitovinany	0.77	0.60
Vakinankaratra	0.92	0.70
Atsimo-Atsinana	0.62	0.05
Atsinanana	0.76	0.39
Ihorombe	0.90	0.23
Boeny	0.86	0.37
Melaky	0.53	0.14
Bongolava	0.63	0.54
Atsimo-Andrefana	0.72	0.11
Diana	0.83	0.41
Anosy	0.83	0.39
Analanjirofo	0.82	0.33
Betsiboka	0.86	0.29
Menabe	0.72	0.25
Androy	0.69	0.09
Sava	0.88	0.41

### 2.3. The burden of congenital rubella syndrome

To project the burden of CRS in Madagascar in each of the 22 regions and evaluate the impact of vaccination programmes, we simulated the age-structured dynamics of rubella in Madagascar (electronic supplementary material, figure S2). Within the simulation, we classified individuals by their age (using monthly age classes up to age 15 and yearly classes thereafter), and epidemiological status (tracking individuals across Maternally immune, Susceptible, Infected, Recovered and Vaccinated classes). We used region-specific birth rates (from www.worldpop.org.uk), and a mortality curve derived from UN projections to capture demographic patterns; we set the generation time of rubella to be two weeks, introduced known patterns of waning of maternal immunity and vaccine efficacy [10], and explored different levels of *R*_0_ (defined as the average number of secondary infections from one infected individual in a completely susceptible population). The range of *R*_0_ values was determined by referencing existing estimates for rubella (ranging from 2 [17] to 12 [18]) and choosing values consistent with the age range of rubella observed in the epidemiological data described above (using the fact that *R*_0_ is approx. equal to *G*/*A*, where *G* is the inverse of the birth rate and *A* is the average age of infection [19]). We also introduced reported levels of routine vaccination coverage for each region for measles from the two sources described above (table 1), and made the conservative assumption that vaccination coverage in Starting Campaigns and Supplementary Immunization Activities (SIA) reaches 60% of the population. The time horizon chosen for evaluation of the burden of CRS and the impact of vaccination was 30 years; we also evaluated the number of years during which the burden of CRS was higher than it would have been at equilibrium pre-vaccination following introduction of the vaccine (transient increases).

### 2.4. Metapopulation dynamics and the burden of congenital rubella syndrome

The simulations described above provide insight into the deterministic burden of CRS. However, previous work indicates that stochastic dynamics within the broader ‘metapopulation’, or network of communities across which rubella persists, or goes extinct and is reintroduced, can result in increased burden in some locations [12]; these same metapopulation processes can drive spatial inequity in changes in the burden of CRS following introduction of the vaccine [11]. Local extinction underpins these changes in CRS burden by allowing susceptible individuals to age into later age classes, and potentially their childbearing years, without exposure to the infection. This leaves them vulnerable to a potential re-introduction of the infection occurring during their pregnancies. Consequently, we also sought to identify areas in Madagascar most likely to be affected by stochastic metapopulation dynamics. To do this, we evaluated the number of districts with population sizes less than the expected Critical Community Size (CCS) for rubella. The CCS describes the minimum population size required to sustain an immunizing infection without stochastic extinction [20], an empirical measure which combines both the theoretical extinction boundary for the infection (assuming a well-mixed population), but also the impact of patterns of re-introduction. While by convention, the CCS is usually framed in terms of total numbers of individuals [20] in reality, the key value will be number of susceptible individuals in a community. Nevertheless, to align with previous work, we focus on total population size and concentrate our evaluations of the CCS only in non-vaccination settings. We focused on districts (smaller administrative units than the regions considered above) in this analysis, because these smaller spatial scales are more likely to represent reasonably well-mixed populations. Evidence from South Africa suggests that the CCS for rubella may be of a similar scale to measles [21], i.e. around 350 000, although data from other countries suggest that it might be closer to 1 000 000 [12,21,22]; simulation approaches suggest that this estimate may be biased upwards [21].

Under-reporting of rubella cases in this dataset precludes direct investigation of the CCS [11], instead we independently evaluated population sizes and their connectivity for districts of Madagascar. With gridded population maps from World Pop (www.worldpop.org.uk), we identified districts with population sizes below the upper the range of previous CCS estimates [11], i.e. 350 000–1 000 000. We then developed expectations for the number of new arrivals in each location based on classic gravity models [23,24]. We used the travel times between centroids of districts as the distance [25–27] using geospatial techniques (see the electronic supplementary material) to develop an accessibility/travel time matrix. Connectivity is measured based on the amount of outgoing travel from each location from the gravity model using travel time distance between district centroids, which yields an index of connectivity for each location (see the electronic supplementary material). More remote areas have fewer trips to other areas of the country. From this, we identified districts that were both weakly connected (remote) and below the CCS threshold, taking both the upper and lower ranges of CCS reported globally. We used stochastic simulations to test expectations for the relationship between connectivity and average age of infection in a district below the CCS (electronic supplementary material, figure S3) finding that more weakly connected locations are expected to show higher variance in the average age of infection. We then plotted average age of infection reported in the incidence data against our index of connectivity to evaluate the degree to which these estimates are able to identify locations at risk of later infection associated with extinction–recolonization dynamics.

### 2.5. Metapopulation dynamics and the potential for rubella extinction in Madagascar

Madagascar is an island and rubella dynamics are predominantly driven by national patterns of incidence, extinction and reintroduction. Data on births and population size for each of the 22 regions of Madagascar, and our TSIR estimates of seasonality in rubella transmission, provide us with the means to simulate stochastic local regional dynamics for rubella. We can extend these simulations to investigate Madagascar-wide metapopulation dynamics by using the estimates of travel magnitude between each of the pairs districts (accessibility matrix) described above. As these measures are relative rather than absolute [25–27], we first rescaled these measures such that they resulted in metapopulation dynamics that reflected estimates of the CCS of rubella in the pre-vaccination era within the range reported for rubella globally (350 000–1 000 000; see the electronic supplementary material, figure S4). Next, we introduced RCV into this framework at rates reported for measles vaccination coverage from administrative coverage in 2014, as well as increased rates, with the aim of identifying the threshold required for rubella elimination in this setting.

## 3. Results

### 3.1. Epidemiology of rubella in Madagascar

Under-reporting in the incidence data affected our ability to estimate the proportion of the population susceptible to rubella (see figure 2*a* and the electronic supplementary material, S1). However, the TSIR model indicated a clear pattern of seasonality in transmission, with low transmission in February, June and October, which was robust to the range of assumptions about starting proportion susceptible (figure 2*b*). Surprisingly, the timing of low transmission does not align with summer school holidays, in contrast to what has been reported for rubella and other immunizing childhood infections in many other parts of the world [22,28,29].

Given low reported incidence (figure 2*a*), we focused our initial descriptive analysis of the epidemiology of rubella in Madagascar at the largest administrative scale available, the six provinces of Madagascar. The average age of infection of rubella is slightly variable across these provinces, ranging from 6.4 years in the province of Antananarivo to 8.8 years in Antsiranana (figure 2*c*). Average age broadly negatively correlates with province population size (Pearson's correlation between log population size and average age of infection yields *ρ* = −0.82, *n* = 6, *p* < 0.05), suggesting an underlying biological driver for this pattern. Two possible drivers of this average infection age distribution include (i) extinction–recolonization dynamics, which drive up the average age of infection in smaller population provinces [12]—implying that provinces with smaller populations also have smaller focal population centres, precluding persistence of rubella or (ii) a higher *R*_0_ for rubella in provinces with larger populations (although see [29] for evidence that measles, a similar directly transmitted infections, shows no signature of density-dependent transmission). With the data available, it is not possible to distinguish between these two possibilities. Using the approximation *R*_0_ = *G*/*A* (which assumes negligible stochastic dynamics), where *G* is the inverse of the birth rate, and *A* is the average age of infection, the average age of infection in the data yields estimates of *R*_0_ between 3.9 in Antsiranana province and 5.5 in Antananarivo province. This range is broadly in line with values previously reported for this infection [15,30,31].

### 3.2. Consequences of introduction of rubella-containing vaccination: deterministic simulations

Simulation of region-specific dynamics built around the estimated profile of seasonal transmission (figure 2*b*) and with *R*_0_ = 5 (see the electronic supplementary material, figure S2 for an example time-series), indicates that in the absence of vaccination, the number of CRS cases varies across the 22 regions of Madagascar between six new CRS cases (in Melaky) and 73 (Analamanga) per year; or between 60 and 81 cases per 100 000 births (figure 3*a*). Introduction of RCV into the routine programme at the lower reported levels of administrative coverage (figure 3*b*) results in a reduction in the burden of CRS in all but five regions (figure 3*c*, Androy, Atsimo-Andrefana, Atsimo-Atsinana, Ihorombe and Melaky); CRS burden is reduced in all regions when the less conservative 2014 estimates of coverage are used (table 1). In both simulations, a number of years of transient outbreaks with a CRS burden higher than would have been observed in the absence of vaccination occur (electronic supplementary material, table S1). If an SIA with 60% coverage is introduced at the same time as the start of routine vaccination against rubella, and maintained at 4 year intervals targeting up to 5 year olds, then the burden of CRS is successfully reduced in all 22 regions (figure 3*d*), and the transient increases in the CRS burden also disappear. Parallel results with *R*_0_ = 8 are shown in the electronic supplementary material, figure S6; for this magnitude of transmission, more regions are at risk of an increase if routine vaccination only is introduced; again, public health consequences can be mitigated by deployment of SIAs. Finally, in this setting, starting campaigns reaching beyond 10 years of age show very little improvement relative to those only extending to 10 years of age (results not shown). The full set of results is shown in the electronic supplementary material, table S1.

### 3.3. The stochastic burden of rubella: exploring risk

**Figure 4.**
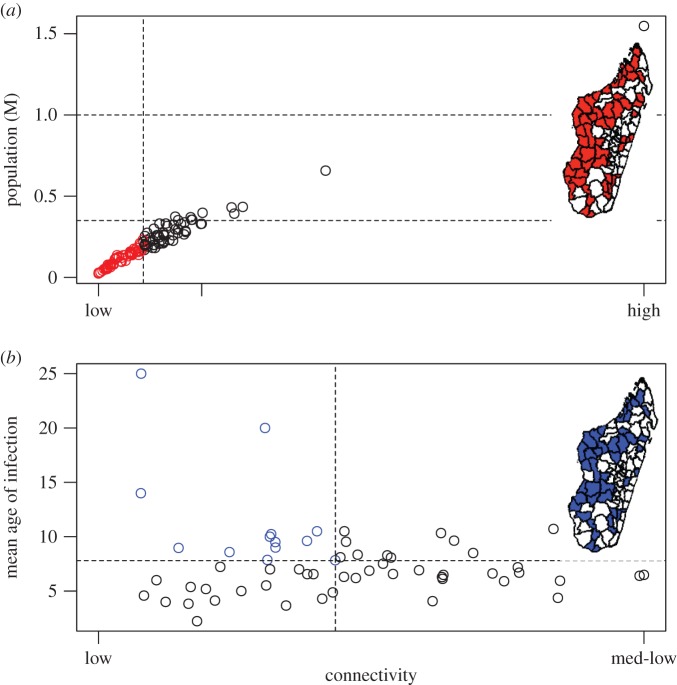
Connectivity, remoteness and rubella dynamics (*a*) District connectivity (*x*-axis) plotted against district population size (*y*-axis) showing communities below the lower limit of the CCS report for rubella (350 000, lower horizontal line), and poorly connected (i.e. dotted line showing the bottom quantile of connectedness, taken to correspond to remote locations) on the figure and the inset map in red. The upper limit of the CCS (1 million) is also shown (higher horizontal line). Connectivity is a relative measure derived from the amount of incoming travel estimated from a gravity model to each district; districts are remote if there is less travel. (*b*) For districts below the CCS, mean age of infection (*y*-axis) plotted against the index of connectivity (*x*-axis), with districts that are weakly connected and below the lower limit of the CCS, but also have experienced a higher average age of infection (and thus might have experienced a higher burden of CRS) shown on the figure and the map in blue.

The majority (54%, 61 out of 114 reporting districts) of districts’ populations in Madagascar are below the lower end of the rubella CCS range (350 000), and all but one are below the upper threshold of 1 million (99%, 113 out of 114 districts). Population size and connectivity are strongly correlated (figure 4*a*) so many districts are both weakly connected (remote) and below both the higher and lower threshold of CCS, shown in figure 4*a* (electronic supplementary material, figure S4*a* indicates the underlying patterns of connectivity), indicating potential for both local extinction, and a long interval before re-introduction of the rubella infection. Although in the simplest analysis, local extinction of an infectious disease seems a desirable outcome, in the context of rubella, principles from metapopulation dynamics suggest that these remote locations below the CCS (depicted on the inset map, figure 4*a*) may have a higher burden of CRS pre-vaccination, as disease-free periods may allow ageing of susceptibles into their childbearing years, leaving them vulnerable to subsequent rubella introductions [12], an issue that may be amplified by inequity in vaccination coverage [21].

One way to evaluate the degree to which our measure of connectivity is dynamically relevant is to evaluate the relationship between average age of infection and remoteness for districts that are below 1 000 000, and thus expected to be vulnerable to stochastic extinction (figure 4*b*). If our connectivity index is capturing a measure of relevance to stochastic extinction, based on simulations of the stochastic dynamics in a location below the CCS (see the electronic supplementary material, figure S3), we expect increased variance in the average age of infection for more remote districts below the upper bound on the CCS. This is broadly what is observed (figure 4) and indicates that higher average age of infection can occur in these less connected districts and potentially poses risks for an elevated burden of CRS. Qualitatively similar results are obtained for the lower threshold on the CCS.

### 3.4. Vaccination and extinction of rubella in Madagascar

Ultimately, the appropriate epidemiological unit for investigating metapopulation dynamics is probably the scale of cities [29]. The closest available scale in this dataset is the districts, as mentioned above. However, in the face of the sparseness of the population in Madagascar, and under-reporting of rubella, in considering the potential for extinction of rubella in Madagascar, we focused on regional scale dynamics. This is equivalent to assuming that each of the 22 regions is reasonably well mixed, and further subdivisions should broadly act to reduce persistence of rubella in Madagascar, by reducing the number of locations above the CCS, so this scale of analysis should be reasonably conservative. Even at this greater spatial scale, the majority of regions (55%, 12 out of 22 regions) have populations that are below the upper end of the CCS range of 1 000 000; a few are also below the threshold for measles of 350 000 (14%, 3 out of 22 regions).

**Figure 5.**
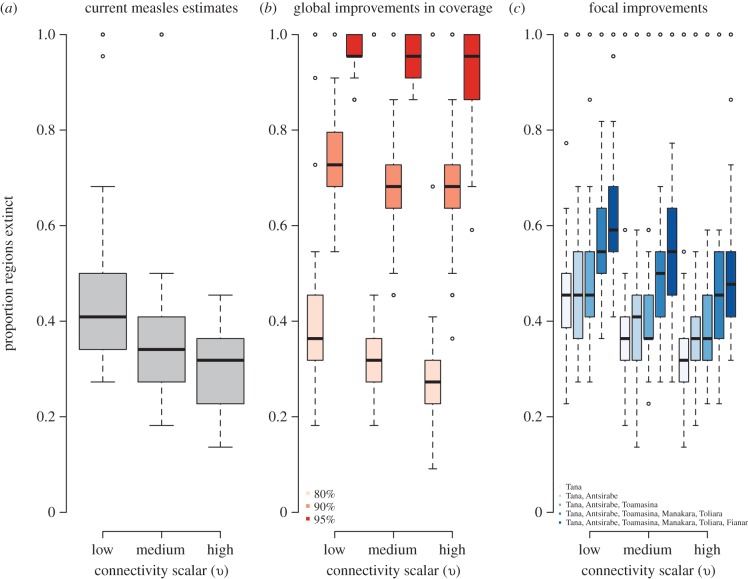
Consequences of vaccination for extinction of rubella in Madagascar showing the proportion of the 22 regions in which rubella is extinct after 20 years of vaccination across 100 simulations for three levels of the connectivity scalar *υ*. This range of values of *υ* were set such that the associated rescaling for the connectivity matrix estimated from geospatial data resulted in dynamics of rubella in Madagascar that captured the globally reported range of the CCS for rubella (see the electronic supplementary material, figure S4) across 100 simulations. Results are shown (*a*) at current reported administrative levels of coverage; (*b*) for 80, 90 and 95% coverage in all regions and (*c*) at reported levels of coverage but with an increase to 95% in focal regions (organized in sequence to explore cumulative effects) whose administrative capitals are indicated in the figure legend. Proportion of simulations in which extinction is achieved is reported in the main text. (Online version in colour.)

Given the isolated nature of Madagascar, low burden of CRS, and increased international and national public health support for control of vaccine-preventable diseases, we analysed the potential for rubella elimination, using simulations of regional dynamics of rubella (see the electronic supplementary material, figures S4 and S5; figure 5). At the current levels of vaccination coverage, the probability of rubella elimination, corresponding to zero rubella incidence in all 22 regions (i.e. points clustered at the top of the bar charts in figure 5*a*) after 20 years of vaccination ranges from 15% (at low levels of connectivity) to 8% (at a high level of connectivity). This probability was estimated as the proportion of simulations (across the 100 simulations) that went extinct in all 22 regions. If vaccination coverage was increased to 90% in all locations, the probability of rubella extinction increases to 39%, regardless of connectivity scalar (from the points clustered at the top of figure 5*b*). If vaccination coverage is increased in focal, well-connected regions only, the probability of rubella elimination moves from 20% at low connectivity and 9% at high connectivity (values clustered at the top of figure 5*c*).

## 4. Discussion

With increased support from the Global Alliance of Vaccines, countries that have previously withheld RCV out of concerns for the potential paradoxical increase in the burden of rubella are now considering introduction of the vaccine. This endeavour presents an opportunity to reduce the burden of a preventable disease (CRS), but may also have additional public health benefits; in particular, that of strengthening existing measles vaccination programmes [32]. Evaluation of the effectiveness of introduction of RCV into a specific country requires an understanding of (i) the current epidemiology of rubella and burden of CRS; (ii) possible areas of increased burden given the landscape of vaccination coverage; and (iii) likely sources and sinks for localized extinction and re-introduction. These factors can be combined to evaluate the effectiveness of vaccination strategies for both routine and supplementary programmes, as well as the potential of rubella elimination.

We evaluated this public health question in the context of Madagascar, a country planning to introduce RCV in the upcoming year (2016). Analysis of rubella data yields results broadly consistent with other descriptions of rubella epidemiology, although the characteristic under-reporting often noted for this infection (which may be so mild as to be asymptomatic) results in considerable uncertainty in some of our inference. In particular, the seasonal pattern detected here does not show strong alignment with school terms (figure 2*b*), in contrast with frequently reported patterns [22,28,33], but uncertainty is sufficiently high such that an alternate pattern (e.g. alignment with agricultural seasons with increased rainfall beginning in October–November [34]) cannot be affirmed with certainty.

A first major concern associated with introduction of RCV is avoidance of the paradoxical effect, i.e. a reduction in rubella incidence insufficient to offset the average age of infection, thus leading to an increase in the burden of CRS [3]. Our age-structured simulation analyses broadly support introduction of RCV in Madagascar, particularly if introduction is accompanied by wide age range campaigns that could be implemented via the existing two forms of additional vaccination opportunities discussed above (SIAs and mother–child health days). When such an additional vaccination campaign is in place, neither long term nor transient increases in the CRS burden, as previously observed in Greece [35] and Costa Rica [36], are observed in simulations for rubella in Madagascar (including simulations with *R*_0_ values as high as 8). These results rest on administrative estimates of routine vaccination coverage. As these estimates are frequently known to be overestimates [16], we also explored scenarios of low routine coverage (table 1) and obtained broadly similar results.

A second major concern associated with introduction of RCV is potential for amplification of inequities associated with the metapopulation dynamics of this infection [11], in particular those associated with heterogeneity in vaccine coverage [37,38]. The deterministic simulations described above ignore the role of stochastic dynamics [12], which can interact with variation in vaccination coverage [11], potentially resulting in local increases in the burden of CRS. To explore these latter issues, we also identified all districts both below the CCS for rubella and weakly connected (remote; figure 4). Variance in the age of incidence broadly increased with diminishing connectivity, suggesting a role for metapopulation dynamics (although spatial variance in *R*_0_ cannot be excluded) and highlighting districts that should be a special focus in evaluating the consequences of introduction of the vaccine. Whatever the underlying cause, districts with higher average age of infection are more at risk of being associated with a higher CRS burden.

Having explored the risks of introduction of RCV into Madagascar, we next investigated positive externalities specific to Madagascar—in particular, the degree to which vaccination against rubella might result in elimination of the virus from the country, a realistic prospect given Madagascar's unique status as an island nation. Assuming that current vaccination coverage estimates reasonably reflect the context in Madagascar, our analysis suggests that the probability of elimination of rubella with introduction of RCV might be as high as 15% over 30 years. Improvements in vaccination coverage, especially targeted at the major hubs of travel (i.e. the most connected areas according to the metrics developed and shown in figure 4) could improve this estimate yet further (figure 5), as well as offer considerable potential for improving measles control. Nevertheless, rubella elimination is not a certainty even with extremely high vaccination coverage across all regions (95%, figure 5). This suggests that targeted vaccination efforts—where vaccine deployment is closely linked to surveillance, either of cases (i.e. as currently employed in outbreak response vaccination for measles [39]), or more powerfully, of serological status within the population—are likely to be more effective components of successful elimination of rubella than blanket vaccination campaigns across broad spatial scales.

Very few measles cases have been reported over Madagascar's fever–rash surveillance system over the past decade, despite regular reports of rubella, suggesting that the system is functioning effectively. These observations also support estimates of administrative coverage, which indicate reasonably high vaccination coverage against measles in Madagascar currently, and over the recent past, thus again bolstering the prospect for elimination of rubella. However, should introduction of RCV into Madagascar be accompanied by the positive outcome of elimination of rubella, the risk of a post-honeymoon outbreak must be an important consideration [40]—the absence of reported cases does not mean that the risk of an outbreak is not building. While rubella elimination will invariably reduce the burden of CRS, there is some sense in which such elimination presents a double edged sword—the absence of circulating cases opens the door to accumulation of susceptible individuals should vaccination coverage not be maintained at high levels. Susceptible build-up is particularly problematic for rubella, as the opportunity for unvaccinated susceptible girls to age into their childbearing years is of major concern. Rubella incidence or control should thus be considered across a long time-horizon [14], with emphasis on maintaining high levels of population immunity via vaccination over the long term.

The availability of rubella data from the pre-vaccination era in Madagascar presents a unique opportunity to evaluate the impact of introduction of RCV into the measles vaccination programme. This opportunity could be further enriched by the improvement of current surveillance, as well as collection of further metrics before, and during, deployment of the vaccine [41]. Some very simple and low-cost extensions of the current system in Madagascar are possible. In particular, mothers whose children present with fever and/or rash are currently questioned about the last vaccine a child has received—with no information about the type of vaccine (e.g. poliomyelitis and measles). Narrowing this question to specifically enquire about measles or measles–rubella vaccine would considerably strengthen this element of reporting. There are also opportunities to design surveys specifically focused on this question—e.g. following previous examples for rotavirus in Malawi [42]. Another question that we did not formally tackle here—and an aspect not currently under consideration in the context of roll-out of the vaccine in Madagascar—is the impact of targeted vaccination of girls or women; previous work has indicated that this may further reduce the risk in an increase in the CRS burden [10] and is a prospect worth considering further in the context of rubella in Madagascar.

**Figure 6.**
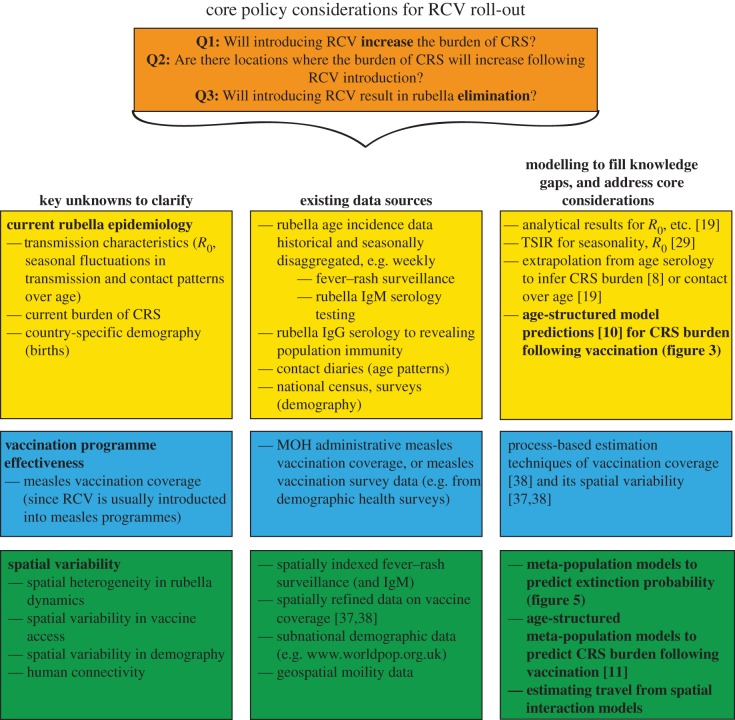
Considerations for roll-out of RCV in low-income settings following opening of the GAVI funding window including key unknowns; existing data sources; and statistical and mathematical approaches to leverage this data, to both identify the key unknowns and tackle the core considerations (shown in bold in the final column). (Online version in colour.)

Here, we lay out the array of core questions for countries considering introduction of RCV following the opening of the GAVI funding window (figure 6), and address them using data from Madagascar. Our analysis indicates that vaccination campaigns will be essential upon introduction of RCV to ensure that long- and short-term increases in the burden of CRS are avoided. It is generally recognized that in the longer term, strengthening of routine programmes and reduced dependence on vaccination campaigns is desirable [43]. If such improvements in routine immunization can be achieved, our results also indicate potential for Madagascar to eliminate rubella over the longer term, especially if targeted vaccination is also deployed. This positive outcome is nevertheless one that will require continued public health vigilance.

## Supplementary Material

Supplementary Information
