# Supplementary material for: Introduction of rubella-containing-vaccine to Madagascar: implications for roll-out and local elimination
Source: J R Soc Interface. 2016 Apr;13(117):20151101. doi: 10.1098/rsif.2015.1101 (PMC4874430; doi:10.1098/rsif.2015.1101)
Supplement: Supplementary Information [file rsif20151101supp1.doc]

**Supplement: Introduction of Rubella-Containing-Vaccine to Madagascar: implications for roll-out and local elimination**

***The TSIR model***

The Time-Series Susceptible-Infected-Recovered model was developed as a method to use simple statistical models to capture discrete time dynamics of childhood infections, building on the simple identity *I_t+1_* = *β I_t_ S_t_* where the time-step is approximately the generation time of the infection, *I_t_* is the number of infected individuals at t, *S_t_*  is the number of susceptibles at time t, and *β* describes transmission from infected to susceptibles. However, deploying this identity first requires that we reconstruct the profile of susceptible individuals through time *S_t_* before estimating transmission (accounting for temporal fluctuations in transmission.) These methodological steps are described in sequence below.

*Susceptible reconstruction*

If reporting rates are stable through time, and all individuals eventually succumb to infection, numbers of susceptible individuals at time t, *S_t_*, in any given location will track births and infected individuals, *I_t_.* The pattern of susceptible individuals through time can then be reconstructed (1, 2) by applying the balance equation:

$S_{t+1}=S_{t}+B_{t}-\frac{I_{t}^{(r)}}{\rho}$, (eq 1)

where *B*_t_ is the number of births (and may be discounted by vaccination where necessary), *ρ* is the reporting rate (here assumed to be constant through space and time) and $I_{t}^{(r)}$ is the reported number of infected cases. Note that in this section on local dynamics we suppress the site-specific subscript for ease of notation. Ignoring observational uncertainty, $I_{t}=I_{t}^{(r)}/\rho$ where *I*_t_ is the actual number of infected individuals at *t*. Rearranging equation 1 provides the relationship from which the reporting rate and the dynamics of the susceptible population can be inferred through susceptible reconstruction:

$S_{t}=\overline{S}N_{t}+D_{0}+\sum_{k=0}^{t-1} B_{k}-\sum_{k=0}^{t-1} \frac{I_{k}^{(r)}}{\rho}$. (eq 2)

where *k* here indicates the time index. In equation 2, $\overline{S}$ represents the average proportion of individuals that are susceptible, *N_t_* represents the population size and *D*_0_ is the unknown deviation around the average at the time of the first observation in the time-series. To estimate *ρ* and reconstruct a full time series of susceptible ‘deviations’, *D_t_*, that details how the numbers of susceptible individuals vary around the average number of susceptible individuals, we write

$\sum_{k=0}^{t-1} B_{k}=D_{t}-D_{0}+\frac{1}{\rho}\sum_{k=0}^{t-1} I_{k}^{(r)}$, (eq 3)

where $D_{t}=S_{t}-\overline{S}N_{t}$. From this, *D_t_* can be estimated as the residuals from the [possibly locally varying] regression of the cumulative number of births on the cumulative number of cases, and *ρ* can be estimated as the inverse slope of this regression (1-3). Note that the average number of susceptible individuals cannot be directly estimated, as it is confounded with the intercept of this regression equation.

*Estimating seasonal transmission*

From this foundation, seasonal transmission rates can be estimated using Time-series Susceptible-Infected-Recovered (TSIR) methods (1, 3). The generation time (serial interval) of rubella (approximately the latent plus infectious period) is approximately 18 days (4, 5), so we assumed that the time-scale of the epidemic process was approximately two weeks, and aggregated the data accordingly. *I*_t+1_ (the number of infected individuals at time t) depends stochastically on *I*_t_ and the number of susceptible individuals *S*_t_ with expectation *E*[(*I_t_*_+1_)] = *β*_s_ *S*_t_ *I*_t_*^m^*/N_t_ where *β*_s_ is the transmission rate in every biweek in any particular location and the exponent *m* (usually a little less than 1) captures heterogeneities in mixing not directly modeled by the seasonality (1, 3) and the effects of discretization of the underlying continuous time process (6). Dividing by *N_t_* captures the fact that social contact networks tend to remain stable with population size (7). Then, taking logs on both side of this relationship, we can write

$\log(E[(I_{t+1})])=\log(\beta_{s})+m\log(I_{t})+\log(D_{t}+\overline{S}N)-\log(N_{t})$. (eq 4)

Given estimates of *I_t_* and D*_t_*, regression techniques can be used to estimate $\beta_{s}$ and *m*, and marginal profile likelihoods can be used to estimate $\bar{S}$ (2, 3). The transmission rate estimated in this way may reflect a broad range of processes that occur consistently over the course of a year, e.g., mixing among school-children (8).

We have previously found that low reporting rates result in strongly downwards-biased estimates of *m*, which result in unrealistic dynamics (8); and this also proved to be the case here. For this analysis, we therefore fixed *m* at a consensus value of 0.97. Previous work (8) indicates that the exact value of *m* does not affect estimates of seasonal variation in transmission. Given the rarity of rubella in many provinces, it was necessary to restrict analysis to the scale of the country. We also found that, even at this scale, profile likelihoods did not allow us to identify the key value of $\bar{S}$, yielding unrealistic values of complete susceptibility (Figure S1B). For further inference, we therefore leveraged the parallel data on average age of infection, alongside estimates from across the African continent (9) to set R_0_=5 and identified the likely starting proportion susceptible via the relationship: $\bar{S}=1/R0$.

**Figure S1: Results from the TSIR analysis** A) Deviance plotted against start proportion susceptible, indicating that no minima is detected across an array of reasonable values; the red vertical line shows the chosen value of$\bar{S}$=0.2, corresponding to R_0_=5, as this is a value consistently found across varied contexts (4), including a recent analysis encompassing an array of countries many on the African Continent (9), and is consistent with estimates of the average age of infection; B) A plot of observed vs. expected incidence for the TSIR model, showing broad agreement for the model fit.

***Age structured simulation for rubella dynamics***

We used a discrete time age-structured framework to model rubella dynamics under various vaccination scenarios in each of the 22 regions of Madagascar. Individuals are classified by age and epidemiological status, which may be as Maternally immune (M), Susceptible (S), Infected (I), Recovered (R) or Vaccinated (V). The key feature of the model is a matrix that at every time-step defines transition from every possible epidemiological stage (M, S, I, R or V) and age combination to every other epidemiological stage and age combination. The time step of the model was set to approximately the generation time of rubella infections, i.e., two weeks, thus there are 24 opportunities to move in and out epidemiologic stages per year. New births also enter into the maternally immune or susceptible class, as appropriate, based on the region specific birth rate, taken from www.worldpop.org.uk. Methods are presented in detail elsewhere (10, 11).

Within the age-structured model we assumed a pattern of contact over age based on diary studies across Europe (12). We set the basic reproductive number (R_0_) for rubella to five, as this is the value returned by a an estimate obtained across multiple African countries (9), as well as being the value reported in a number of other contexts (4); we also present results for R_0_=8 as this has been shown to be linked to potential problems subsequent to introduction of RCV (10).

Model simulations were initiated in the year 2000 using the demographic structure inferred for that year; and propagated forward until 2015 to remove transient infectious disease dynamics while maintaining appropriate population structure. Dynamics were then followed for a further 30 years under varying vaccination scenarios, including i) no vaccination; ii) routine vaccination only (with coverage levels taken to reflect estimates for the focal region); iii) a starting campaign up to age 10 at 60% coverage followed by campaigns at 4 year intervals targeting children aged between one and five at 60% coverage; iv) likewise but with the starting campaign reaching up to age 15; v) or 20; vi) or 25. Results for incidence and average age of infection are shown in Figure S2A and B.

The CRS burden was evaluated taking the product 0.5 x **I_a_** x *f_a_* x 9/12 x 1/3 x 0·65, where **I_a_** is the total number infected in age class *a* in a year, and 0.5 restricts this to the female half of the populations; *f_a_* is the fertility in that year, the two fractions capture the fact that pregnancy lasts 9 months of the year; and the women are only vulnerable to adverse outcomes via CRS in the first trimester; finally, 0·65 is the probability of adverse outcomes following rubella infection during the first trimester of pregnancy (13). Corresponding estimates of fluctuations in CRS burden are shown in Figure S2C.

**Figure S2: An illustration of the deterministic simulation results for the region of Analamanga** indicating A) incidence through time, B) average age of infection, and C) the number of cases of CRS. The colors and line widths distinguish different scenarios. If the population remains unvaccinated (black line) incidence (A) follows characteristic seasonal fluctuations, and average age (B) and CRS burden (C) remain broadly constant. For the scenario where routine immunization only is introduced (grey line) incidence falls gently, and average age rises; nevertheless, the fall in incidence is sufficient that this results in a decline in the CRS burden (bottom panel); and after about 15 years, population immunity reaches sufficiently high levels that rubella circulation is negligible if routine vaccination is maintained. For the scenario where vaccination is implemented via routine immunization with a starting campaign up to 10 years old on introduction (thin red line), incidence falls sharply as does CRS burden; thicker red lines indicate increasing age range of starting campaigns to 15, 20 and 25 years old.

***Estimating an index of connectivity for each district of Madagascar***

To characterize the degree of connectivity of districts across Madagascar, we used an unparameterized gravity model based on the populations of the origin (
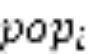
), destination
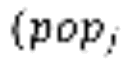
),, and a measure of distance (
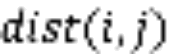
), between these locations.


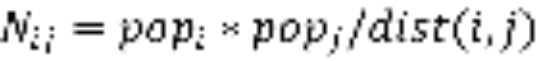


To calculate the distance measure, we combined road networks data (http://www.openstreetmap.org/), land cover (http://due.esrin.esa.int/page_globcover.php), and topography ([http://srtm.csi.cgiar.org/](https://owa.princeton.edu/owa/redir.aspx?SURL=BqNCGEuJl6j6sLoar7F1tWQCf0urDHYdIE3h7b4hZcFDS3PVJtrSCGgAdAB0AHAAOgAvAC8AcwByAHQAbQAuAGMAcwBpAC4AYwBnAGkAYQByAC4AbwByAGcALwA.&URL=http%3A%2F%2Fsrtm.csi.cgiar.org%2F)), using methods described in detail in (14) to produce an ‘access’ distance matrix. This is derived by first developing an estimate of ‘friction’ between one location and another, a quantity that takes into account land cover types, transport networks and gradient, and is generally thought to be more representative measure of ease of human travel across a landscape than simple linear distance, as it reflects impedances to travel. The friction surface can then be converted to a map of estimated travel times to features of interest. From this, electrical circuit theory can be used, to calculate ‘access’ distances between sample locations across the friction surface: the isolation by resistance model uses a graph theoretic distance metric based on circuit theory to simultaneously consider all possible pathways connecting sample location pairs: the result is a matrix of access distances across the friction surface from each sampled location to every other sample location. These distance measures were used in our gravity model. To estimate the amount of connectivity for each district, we used calculated the total number of outgoing trips from each district. This produced our measure of connectivity for each location.

***Expectations for the average and variance in the age of infection in the least connected (most remote) communities below the Critical Community Size***

To evaluate whether a signature of remoteness could be detected in the age profile of rubella infection of a district, we used the age-structured model described above to simulate the stochastic dynamics of rubella in a location below the CCS, i.e., subject to regular extinctions. We used the demographic rates of the region of Atsimo-Andrefana, with 0.75 vaccination coverage to bring susceptible replenishment levels below the CCS; reframed the model to capture stochastic variation in all rates, and introduced a parameter describing the influx of infected individuals, which was simulated by taking draws from a binomial distribution. With this, we simulated 5 replicates of 30 years of the resulting dynamics of rubella, across a gradient from 1 to 50 infected individuals arriving every year. At lower rates of introduction of infected individuals (likely to characterize weakly connected communities) the age of infection shows greater variability (Figure S3). Districts in Madagascar that we evaluated as being below the CCS showed a similar pattern (Figure 4, main text)

**Figure S3:** Relationship between A) mean (y axis) or B) variance (y axis) in the age of infection for stochastic simulations of a community below the CCS as a function of the rate of infected individuals arriving each year (x axis), an index of community remoteness. Associated time-series are illustrated on the bottom panel; each showing 5 simulations (indicated in different colours) ranging from a C) low, D) medium and E) highly connected communities.

***Patterns of travel and metapopulation dynamics of rubella***

Estimates of relative magnitude of connectivity between each of the regions of Madagascar based on a gravity model combined with travel time (15) are shown in Figure S4A. Analamanga, the region in the center of the country containing the capital city, Antananarivo, dominates both travel to and from other regions; followed by the region of Vakinankaratra just to its south, which contains the important city of Antsirabe. We next identified the range of values of a scalar *υ* such that the influx of infected individuals defined by the product of this value and the matrix of connectivity **C** resulted in metapopulation dynamics associated with a CCS within the range reported for rubella (see text).

The metapopulation of rubella across the regions of Madagascar was simulated assuming that the numbers of new infected individuals in each region *j* followed a negative binomial distribution with mean $\beta_{t}I_{j,t}^{m}S_{j,t}/N_{j}$ and variance *I_j,t_*  (3). In this expression, *β_t_* is the seasonal transmission rate estimated by the TSIR, *I_j,t_* and *S_j,t_* are the numbers of infected and susceptible individuals in region *j* at time step t respectively, and *N_j_* is the population size of the *j*^th^ region. The exponent *m*, usually a little less than 1, captures heterogeneities in mixing not directly modelled by the seasonality (3) and the effects of discretization of the underlying continuous time process (6). The number of new susceptibles follows *S_j,t_* = S*_j,t-1_* + B*_j_* - I *_j,t_* where *S_j,t_* and *I_j,t_* are as above, and *B_j_* is the number of births in region *j* in one biweek. The vector that sets the probability of an infected immigrant arriving into each region is *υ***C*I****_t_* where **C** is the matrix of connectivity (Figure S4A), *υ* is a scalar, and ***I****_t_* is the vector describing the number of infected individuals in each region at time step *t* according to the regional dynamics described above. This is used to generate deviates from a binomial distribution, which determine the arrival, or otherwise, of a new infected immigrant.

For increasing values of *υ*, we simulated the metapopulation dynamics 100 times, and identified the proportion of times that zero cases were reported for each region after a burn in period during which extinction was prevented. We then fitted the median proportion of zeros as the response variable in a binomial regression with log population size of each region as the covariate (Figure S4B). The CCS was defined as the smallest population size for which <0·05% of the time-series was predicted to be zero. This value declines with the overall magnitude of connectivity shaped by *υ* (Figure S4C); values of *υ* between 8·9e-06 and 1·5e-05 result in the reported range of the CCS for rubella.

***Consequences of vaccination in the metapopulation***

To explore requirements for rubella extinction in Madagascar, we simulated the metapopulation in the absence of vaccination, and then introduced vaccination into each of the regions, exploring both reported levels of measles vaccination (time-series shown in Figure S5, results in Figure 5A), the situation if 80%, 90% or 95% vaccination coverage of the birth cohort could be achieved in all regions (Figure 5B), and if 95% vaccination coverage could be achieved in only the largest and most connected regions (Figure 5C) across the range of connectivity identified as relevant (see above).

**Figure S4: Simulating the metapopulation of regions of Madagascar** A) Connectivity between each of the regions of Madagascar was obtained as described in the text; B) Log region population size (x axis) is plotted against the median proportion of zeros in each region (y axis, black points) across one hundred simulations of the metapopulation dynamics (quartiles shown in grey) associated with increasing magnitude of connectivity scaled by *υ* (rows). A negative binomial model was fitted to this data (blue line) to identify the CCS (see text); this was compared to the reported range (red arrows). C) Increasing values of *υ* (x axis from 4e-6 to 1.8e-5) result in declining estimates of the CCS (y axis) as expected: rubella should be able to persist at smaller population sizes in more highly connected populations where metapopulation rescue is more likely. The range of values of *υ* that match the reported range (shaded red area) ranges from 9·5e-6 to 1·5e-5, and this was used to explore the consequences of vaccination.

**Figure S5: Simulated metapopulation of rubella in Madagascar** showing biweekly case counts for each of the 22 regions, with introduction of vaccination at levels reported for measles in the 21^st^ year of the simulation (vertical red line).

**Figure S6: The average annual burden of Congenital Rubella Syndrome** **for R_0_=8** A) Simulated average annual burden in the absence of vaccination; B) Simulated deterministic average annual burden of CRS over 30 years following introduction of routine vaccination only, using the conservative scale (i.e., 2015 values, see Table 1); C) Simulated deterministic average annual burden of CRS over 30 years following introduction of routine vaccination with a starting campaign reaching up to age 10 with 60% coverage, and follow-up campaigns to age 5 occurring every 4 years. See Table S1 for the full set of results. D) Simulated deterministic average annual burden of CRS over 30 years following introduction of routine vaccination with a starting campaign reaching up to age 10 and a follow-up campaign to age five occurring every four years. Both campaigns have a coverage of 60%. See Table S1 for the full set of results.


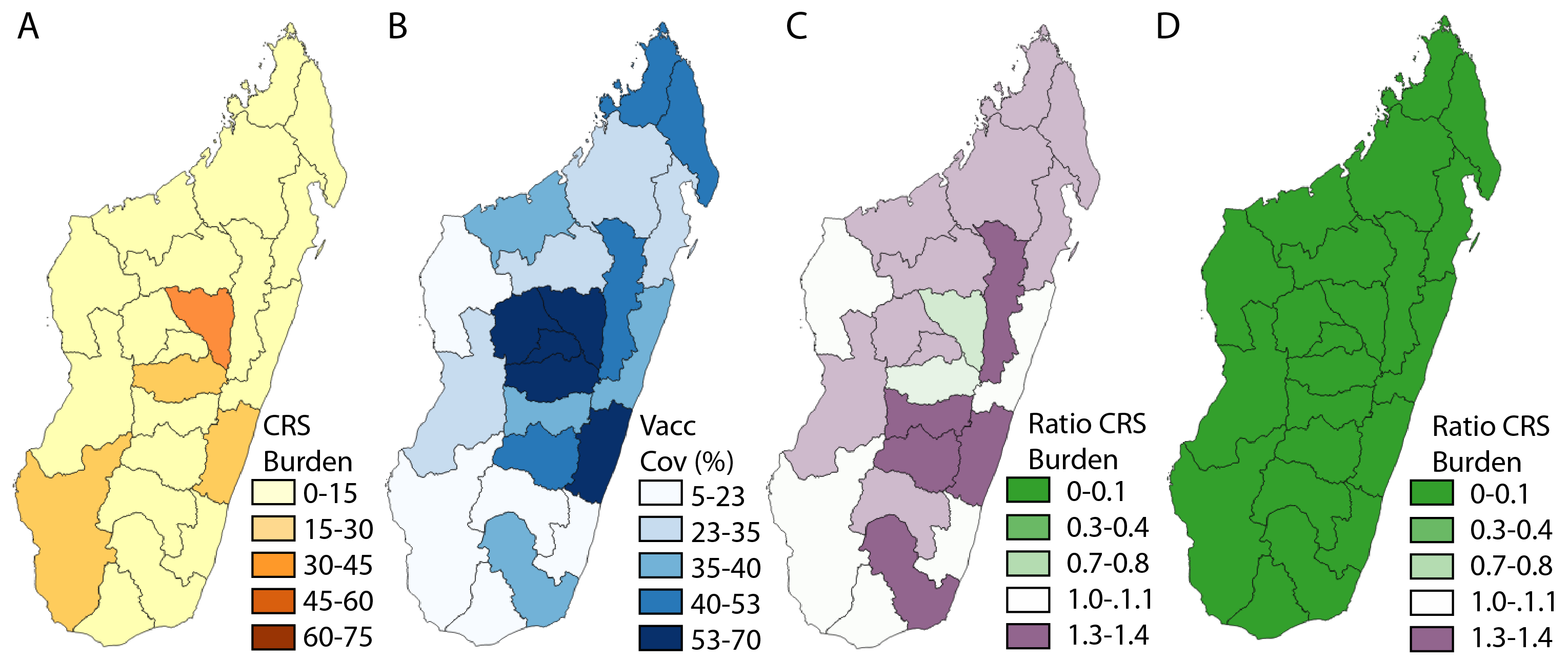


**Table S1: Results of deterministic simulations of the average annual burden of CRS for the 22 regions of Madagascar over 30 years for R_0_=5 (first rows) and R_0_=8 (subsequent rows); and consequences of introduction of RCV into Madagascar for conservative (columns 3-6) and current administrative estimates (columns 7-10) of vaccination coverage; see Table 1 in the main text for vaccination coverage values used.**

|  |  | **Conservative vaccination coverage (2015 estimates)** | | | | **Administrative vaccination coverage (2014)** | | | |
| --- | --- | --- | --- | --- | --- | --- | --- | --- | --- |
| **Region** | **CRS burden** | **Ratio with only routine** | **Ratio routine + SIA and starting campaign to age 10** | **Number of years showing a transient CRS increase, routine only** | **Number of years showing a transient CRS increase, routine + SIA and starting campaign to age 10** | **Ratio with only routine** | **Ratio routine + SIA and starting campaign to age 10** | **Number of years showing a transient CRS increase, routine only** | **Number of years showing a transient CRS increase, routine + SIA and starting campaign to age 10** |
| **Results for R_0_=5** |  |  |  |  |  |  |  |  |  |
| Alaotra-Mangoro | 25·63 | 0·86 | 0·00 | 0 | 0 | 0·15 | 0·00 | 0 | 0 |
| Amoron'i | 18·38 | 0·93 | 0·00 | 0 | 0 | 0·13 | 0·00 | 0 | 0 |
| Analamanga | 72·44 | 0·22 | 0·01 | 0 | 0 | 0·15 | 0·01 | 0 | 0 |
| Analanjirofo | 23·49 | 0·93 | 0·01 | 7 | 0 | 0·13 | 0·01 | 0 | 0 |
| Androy | 17·18 | 1·01 | 0·00 | 18 | 0 | 0·21 | 0·00 | 0 | 0 |
| Anosy | 16·84 | 0·92 | 0·00 | 0 | 0 | 0·12 | 0·00 | 0 | 0 |
| Atsimo-Andrefana | 37·56 | 1·01 | 0·00 | 20 | 0 | 0·15 | 0·00 | 0 | 0 |
| Atsimo-Atsinana | 24·34 | 1·01 | 0·00 | 20 | 0 | 0·48 | 0·00 | 0 | 0 |
| Atsinanana | 24·19 | 0·82 | 0·01 | 0 | 0 | 0·15 | 0·01 | 0 | 0 |
| Betsiboka | 7·03 | 0·99 | 0·00 | 18 | 0 | 0·11 | 0·00 | 0 | 0 |
| Boeny | 18·38 | 0·92 | 0·01 | 0 | 0 | 0·12 | 0·01 | 0 | 0 |
| Bongolava | 8·94 | 0·63 | 0·01 | 0 | 0 | 0·36 | 0·01 | 0 | 0 |
| Diana | 14·79 | 0·85 | 0·01 | 0 | 0 | 0·13 | 0·01 | 0 | 0 |
| Haute matsiatra | 36·35 | 0·87 | 0·00 | 0 | 0 | 0·11 | 0·00 | 0 | 0 |
| Ihorombe | 8·87 | 1·04 | 0·00 | 20 | 0 | 0·09 | 0·00 | 0 | 0 |
| Itasy | 18·36 | 0·37 | 0·00 | 0 | 0 | 0·27 | 0·00 | 0 | 0 |
| Melaky | 6·12 | 1·00 | 0·01 | 18 | 0 | 0·71 | 0·01 | 0 | 0 |
| Menabe | 14·09 | 0·99 | 0·01 | 18 | 0 | 0·16 | 0·00 | 0 | 0 |
| Sava | 22·93 | 0·88 | 0·01 | 0 | 0 | 0·11 | 0·01 | 0 | 0 |
| Sofia | 27·04 | 0·96 | 0·01 | 13 | 0 | 0·11 | 0·01 | 0 | 0 |
| Vakinankaratra | 46·69 | 0·17 | 0·00 | 0 | 0 | 0·10 | 0·00 | 0 | 0 |
| Vatovavy Fitovinany | 40·49 | 0·56 | 0·00 | 0 | 0 | 0·13 | 0·00 | 0 | 0 |
| **Results for R_0_=8** |  |  |  |  |  |  |  |  |  |
| Alaotra-Mangoro | 10·65 | 1·24 | 0·00 | 20 | 0 | 0·77 | 0·00 | 0 | 0 |
| Amoron'i | 7·56 | 1·23 | 0·00 | 20 | 0 | 0·55 | 0·00 | 0 | 0 |
| Analamanga | 33·25 | 0·86 | 0·01 | 7 | 0 | 0·29 | 0·01 | 0 | 0 |
| Analanjirofo | 10·09 | 1·15 | 0·00 | 20 | 0 | 0·16 | 0·00 | 0 | 0 |
| Androy | 7·14 | 1·05 | 0·00 | 22 | 0 | 0·99 | 0·00 | 18 | 0 |
| Anosy | 6·99 | 1·22 | 0·00 | 20 | 0 | 0·16 | 0·00 | 0 | 0 |
| Atsimo-Andrefana | 15·26 | 1·07 | 0·00 | 22 | 0 | 0·90 | 0·00 | 7 | 0 |
| Atsimo-Atsinana | 9·82 | 1·03 | 0·00 | 22 | 0 | 1·23 | 0·00 | 20 | 0 |
| Atsinanana | 11·21 | 1·09 | 0·01 | 19 | 0 | 0·34 | 0·01 | 0 | 0 |
| Betsiboka | 2·87 | 1·18 | 0·00 | 22 | 0 | 0·13 | 0·00 | 0 | 0 |
| Boeny | 7·79 | 1·18 | 0·00 | 20 | 0 | 0·13 | 0·00 | 0 | 0 |
| Bongolava | 3·94 | 1·12 | 0·01 | 19 | 0 | 1·01 | 0·01 | 18 | 0 |
| Diana | 6·48 | 1·15 | 0·01 | 20 | 0 | 0·15 | 0·01 | 0 | 0 |
| Haute matsiatra | 14·45 | 1·32 | 0·00 | 21 | 0 | 0·26 | 0·00 | 0 | 0 |
| Ihorombe | 3·43 | 1·18 | 0·00 | 22 | 0 | 0·10 | 0·00 | 0 | 0 |
| Itasy | 7·51 | 1·14 | 0·00 | 19 | 0 | 1·08 | 0·00 | 19 | 0 |
| Melaky | 2·59 | 1·07 | 0·01 | 21 | 0 | 1·20 | 0·01 | 20 | 0 |
| Menabe | 5·91 | 1·13 | 0·00 | 21 | 0 | 0·85 | 0·00 | 3 | 0 |
| Sava | 9·73 | 1·19 | 0·00 | 20 | 0 | 0·12 | 0·00 | 0 | 0 |
| Sofia | 11·82 | 1·11 | 0·01 | 21 | 0 | 0·12 | 0·00 | 0 | 0 |
| Vakinankaratra | 19·52 | 0·91 | 0·00 | 15 | 0 | 0·10 | 0·00 | 0 | 0 |
| Vatovavy Fitovinany | 16·36 | 1·26 | 0·00 | 20 | 0 | 0·60 | 0·00 | 0 | 0 |

**References**

1. Finkenstadt B, Grenfell BT. Time series modelling of childhood diseases: a dynamical systems approach. Journal of the Royal Statistical Society, Series C. 2000;49:187-205.

2. Finkenstadt B, Bjørnstad ON, Grenfell BT. A stochastic model for extinction and recurrence of epidemics: estimation and inference for measles outbreaks. . Biostatistics. 2002;3:493-510.

3. Bjørnstad ON, Finkenstadt B, Grenfell BT. Endemic and epidemic dynamics of measles: Estimating epidemiological scaling with a time series SIR model. Ecological Monographs. 2002;72:169-84.

4. Anderson RM, May RM. Infectious diseases of humans. Oxford, OX2 6PD: Oxford University Press; 1991.

5. Banatvala JE, Brown DWG. Rubella. Lancet. 2004;363:1127-37.

6. Glass K, Xia Y, Grenfell BT. Interpreting time-series analyses for continuous-time biological models-measles as a case study. Journal of Theoretical Biology. 2003;223:19-25.

7. Ferrari MJ, Perkins S, Pomeroy L, Bjornstad ON. Pathogens, social networks and the paradox of transmission scaling. Interdisciplinary Perspectives on Infectious Diseases. 2011: ID 267049.

8. Metcalf CJE, Bjørnstad ON, Grenfell BT, Andreasen V. Seasonality and comparative dynamics of six childhood infections in pre-vaccination Copenhagen. Proceedings of the Royal Society of London, Series B. 2009;276:4111-8

9. Lessler J, Metcalf CJE. Balancing evidence and uncertainty when considering rubella vaccine introduction. PloS One. 2013;8:e67639.

10. Metcalf CJE, Lessler J, Klepac P, Cutts FT, Grenfell BT. Minimum levels of coverage needed for rubella vaccination: impact of local demography, seasonality and population heterogeneity. Epidemiology and Infection. 2012;16:1-12.

11. Metcalf CJE, Lessler J, Klepac P, Morice A, Grenfell BT, Bjornstad ON. Structured models of infectious disease: inference with discrete data. Theoretical Population Biology. 2012;82 (4):275-82.

12. Mossong J, Hens N, Jit M, Beutels P, Aranen K, Mikolajczyk R, et al. Social Contacts and Mixing Patterns Relevant to the Spread of Infectious Diseases. PloS Medicine. 2008;5:e74.

13. Vynnycky E, Gay NJ, Cutts FT. The predicted impact of private sector MMR vaccination on the burden of Congenital Rubella Syndrome. Vaccine. 2003;21:2708-19.

14. Tatem AJ, Hemelaar J, Gray RR, Salemi M. Spatial accessibility and the spread of HIV-1 subtypes and recombinants. Aids. 2012;26(18):2351-60.

15. Linard C, Gilbert M, Snow RW, Noor AM, Tatem AJ. Population Distribution, Settlement Patterns and Accessibility across Africa in 2010. PloS One. 2012;7:e31743. .
